# A Modified Viscoelastic Point-of-Care Method for Rapid Quantitative Detection of Enoxaparin: A Single-Centre Observational Study

**DOI:** 10.3390/jcm14041328

**Published:** 2025-02-17

**Authors:** Endre Hajdu, Eva Molnar, Katalin Razso, Agota Schlammadinger, Anita Arokszallasi, Csenge Greta Lukacs, Bela Fulesdi, Zsuzsanna Bereczky, Zsolt Olah

**Affiliations:** 1 Department of Anesthesiology and Critical Care, Faculty of Medicine, University of Debrecen, Nagyerdei blvd 98, 4032 Debrecen, Hungaryfulesdi@med.unideb.hu (B.F.); 2 Division of Clinical Laboratory Science, Department of Laboratory Medicine, Faculty of Medicine, University of Debrecen, Nagyerdei blvd 98, 4032 Debrecen, Hungary; molnare@med.unideb.hu; 3 Division of Hematology, Department of Internal Medicine, Faculty of Medicine, University Of Debrecen, Nagyerdei blvd 98, 4032 Debrecen, Hungary; 4 Department of Oncology, Faculty of Medicine, University Of Debrecen, Nagyerdei blvd 98, 4032 Debrecen, Hungary

**Keywords:** hemostasis, low-molecular-weight heparin monitoring, viscoelastometric testing, bleeding, point-of-care testing

## Abstract

**Background:** Laboratory monitoring of the effect of low-molecular-weight heparins (LMWHs) is generally not necessary. However, prompt evaluation of heparin inhibitory effects (i.e., anti-Xa activity) is important in cases of life-threatening bleeding, need for urgent surgery or acute thromboembolism under LMWH treatment. We aimed to establish a simple and reliable point-of-care method for the detection of enoxaparin. **Methods:** Eighty patients under enoxaparin therapy and ten healthy volunteers without any anticoagulant treatment were enrolled. Simultaneous measurements of anti-Xa activity using the chromogenic method and clotting times in the absence and presence of polybrene using viscoelastometric assays containing Russell’s viper venom (RVV-test) were performed on the ClotPro device. **Results:** Among the measured and derived RVV-test parameters, the ratio of the RVV clotting times (RVV CT) detected in the absence and presence of polybrene showed the best statistically significant correlation with anti-Xa activity (r = 0.774, *p* < 0.001). Based on ROC analysis, we designated RVV CT ratios of 1.02, 1.23 and 1.6 as the best cut-off values for separating anti-Xa ranges below and above 0.3 and 0.6 IU/mL, respectively. If the RVV CT ratio is below or above 1.23, the anti-Xa activity is suggested to be below 0.6 IU/mL or above 0.3 IU/mL with high certainty, respectively. Further differentiation is possible if the RVV CT ratio is measured below 1.02 or above 1.6. In these cases, the measured anti-Xa values are below 0.3 IU/mL or above 0.6 IU/mL, respectively, with high probability and good predictive values. **Conclusions:** Our method can provide semiquantitative information on the effect of enoxaparin and the expected anti-Xa activity within 10 min in real clinical situations.

## 1. Introduction

Low-molecular-weight heparin (LMWH) is a widely used therapy for thromboprophylaxis, arterial and venous thromboembolism. It has a particularly prominent role in perioperative settings, pregnancy, malignant diseases and the acute phase of arterial and venous thromboembolism [1,2,3,4,5]. It has a mainly Factor Xa (Xa) inhibitory effect with a relatively predictable pharmacokinetics and dose–response relationship. Therefore, routine monitoring of its effect is generally not necessary [6]. However, monitoring may be required in certain situations, such as renal insufficiency, extreme body weight, pregnancy and patients with high bleeding risk [7,8]. In addition, prompt evaluation of anti-Xa activity is particularly useful in cases of life-threatening bleeding, need for urgent surgery or acute thromboembolism supporting key decisions, such as the use of an antidote, postponement of surgery or dose modification. LMWHs do not have a consistent or reliable effect on traditional coagulation assays like the Prothrombin Time (PT) and Activated Partial Thromboplastin Time (aPTT) assays [9,10]. Nowadays, the gold standard method for the measurement of the activity of LMWHs is based on chromogenic anti-Xa assays [11,12].

Although chromogenic anti-Xa testing is used in some centres with a short turnaround time (TAT) of about 30 min, its availability in a 24/7 system may be less widespread [13,14]. Furthermore, in urgent clinical cases with a serious time factor, even the 30 min TAT may be more than expected. The point-of-care viscoelastometric tests (VETs) can provide more rapid, comprehensive and real-time information on coagulation, clot formation and fibrinolysis by testing the whole blood. The time required for the development of an initial detectable clot is called reaction time (R) or clotting time (CT) depending on the type of viscoelastometric method used [15,16]. The fact that anticoagulants variably prolong these parameters led to many attempts to quantify LMWHs by the most commonly used VETs, such as thrombelastography (TEG) or rotational thrombelastometry (ROTEM). However, these attempts have failed so far in real clinical conditions [10,17,18,19,20].

ClotPro is a viscoelastometric device with some specific reagents including the RVV-test, which contains the direct FX activator Russell’s viper venom (RVV). Based on their measurements in COVID-19 patients, Bösch J. et al. suggested a cut-off of 78 and 120 s of clotting time in the RVV-test for anti-Xa activities below 0.3 IU/mL and above 0.5 IU/mL, respectively [21]. However, clotting times obtained by these methods are influenced by many factors; hence, their cut-off values were not applicable in our patients due to weak sensitivity, specificity and predictive values in our everyday practice.

Therefore, taking into account the experiences of the previously published studies, we aimed to establish a simple and reliable point-of-care (POC) method for the detection of enoxaparin, one of the most commonly used LMWHs.

## 2. Materials and Methods

### 2.1. Study Setting

After obtaining ethics committee approval of the Regional Institutional Research Ethics Committee (DE RKEB/IKEB Ethical Committee), Clinical Centre, University of Debrecen, Hungary (Permission ID: 5926-2021) on 15 December 2021, we conducted a single-centre real-life clinical laboratory investigation between June 2023 and December 2023.

### 2.2. Patients

Patients admitted to University of Debrecen, Hungary, above age 18 and receiving treatment with enoxaparin, regardless of the indication, were eligible for the study. Patients on treatment with any other or more than one anticoagulant were excluded. The control group consisted of ten healthy volunteers without any known haemostatic abnormality and without enoxaparin or other anticoagulant treatment. Data of baseline patient characteristics and indications of enoxaparin were collected. Enoxaparin was given subcutaneously at a dose prescribed by the attending physician.

### 2.3. Study Outcomes

**Figure 1:**
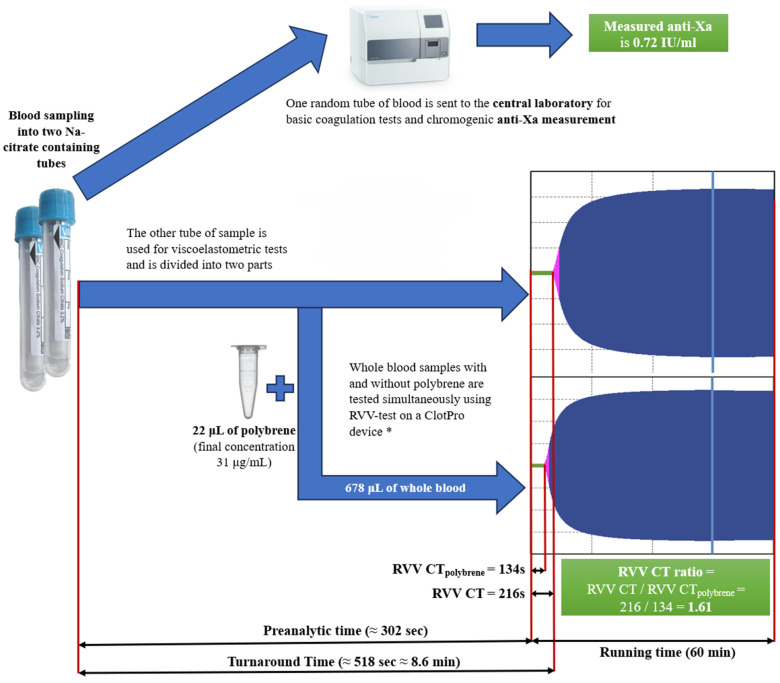
Workflow summary illustrated by a real measurement. In this real case, the RVV CT ratio is 1.61, being above 1.6; therefore, the estimated anti-Xa is above 0.6 IU/mL. The measured anti-Xa activity using the chromogenic method is 0.72. Turnaround time = preanalytic time (≈302 s) + longer duration of the RVV CTs measured in the presence and absence of polybrene (134 s < 216 s) = 302 + 216 = 518 s ≈ 8.6 min. * The CloPro device has 6 independent channels, so 6 measurements can be performed in parallel at the same time.

Blood samples were collected and processed according to the recommendations of the International Council for Standardisation in Haematology [22,23]. Assuming a real emergency clinical situation, the timing of blood sampling was not strictly dependent on the time of administration of enoxaparin and recording of the hours elapsed between them was not required. Samples were collected in Vacuette^®^ tubes (Greiner Bio-One, Kremsmünster, Austria) containing 3.2% buffered trisodium citrate, with a citrate:blood volume ratio of 1:9. Two tubes of blood were collected from each subject. One random tube of blood was sent to the central laboratory for basic coagulation tests and chromogenic anti-Xa measurement (Innovance Heparin, Siemens, Marburg, Germany) on a BCS-XP automated coagulometer (Siemens, Marburg, Germany). The other tube of sample was used for viscoelastometric tests and divided into two parts. One portion of 678 microliters of whole blood was separated, to which 22 microliters of polybrene (hexadimethrine bromide, Merck, Darmstadt, Germany) was added, resulting in a sample with a final concentration of 31 μg/mL polybrene. The second portion of blood remained free from polybrene. Whole blood samples with and without polybrene were tested simultaneously using a ClotPro device (Enicor GmbH, Munich, Germany) within 30 min after blood sampling, under standardised conditions (37 °C, running time 60 min) (Figure 1). RVV assay (Enicor GmbH, Munich, Germany) was performed on each sample according to the manufacturer’s instructions and basic parameters of each VET, such as clotting time (CT), alfa angle, clot formation time (CFT), amplitudes at 5, 10 and 20 min after the end of clotting time (A5, A10, A20) and maximal clot firmness (MCF), were collected. Additionally, delta CT and RVV CT ratios were calculated from the clotting times measured in the presence (RVV CTpolybrene) and in the absence of polybrene (RVV CT) according to the following formula: Delta CT = RVV CT − RVV CTpolybrene; RVV CT ratio = RVV CT/RVV CTpolybrene. Quality controls were performed according to the standards of manufacturers.

### 2.4. Statistical Analysis

Correlation was investigated between the anti-Xa activity and basic viscoelastometric or its derived parameters, such as RVV CT, RVV delta CT, RVV CT ratio, alfa angle, CFT, A5, A10, A20 and MCF. Distribution of continuous variables was investigated using the Kolmogorov–Smirnov and Shapiro–Wilk tests. Continuous variables were expressed as mean and Standard Deviation (SD) in cases of normal distribution or as median, minimum–maximum values and interquartile ranges (IQR) in cases of non-Gaussian distribution. IQR is the region between the 25th–75th percentile. Pearson or Spearman bivariate correlations were performed according to normal or non-normal distribution, respectively. Differences between continuous variables were investigated by Student’s *t*-test or ANOVA in cases of normal distribution and by Mann–Whitney U tests or Kruskal–Wallis tests in cases of non-Gaussian distribution. Receiver operating characteristic curves (ROC) for the derived ClotPro parameters were calculated for anti-Xa values of 0.3 IU/mL and 0.6 IU/mL; specificity, sensitivity and predictive values were also calculated. A *p* value < 0.05 was considered as statistically significant. Statistical analysis was performed by using SPSS 28.0 (IBM Corp., Armonk, NY, USA).

## 3. Results

Eighty patients and ten healthy volunteers were enrolled into the study from June 2023 to December 2023. A total of 180 blood samples were obtained and simultaneous measurements of anti-Xa activity and ClotPro RVV assays were performed with and without the use of polybrene. The basic characteristics of the patients, the indications of anticoagulation, anti-Xa activities and clotting times of ClotPro RVV assays are shown in Table 1.

Among the screening tests of coagulation, the distribution of prothrombin time and thrombin time was non-normal, while aPTT showed a normal distribution. Thrombin time showed a significant, but relatively weak, correlation with anti-Xa values (r = 0.567, *p* < 0.001). Correlation coefficients and *p*-values for prothrombin time and aPTT were r = −0.113, *p* = 0.399 and r = 0.004, *p* = 0.979, respectively.

Regarding the distribution of anti-Xa values, the CT, CFT and alpha angles in the presence and absence of polybrene were non-Gaussian, while other ClotPro parameters including A5, A10, A20 and MCF showed normal distribution.

**Figure 2:**
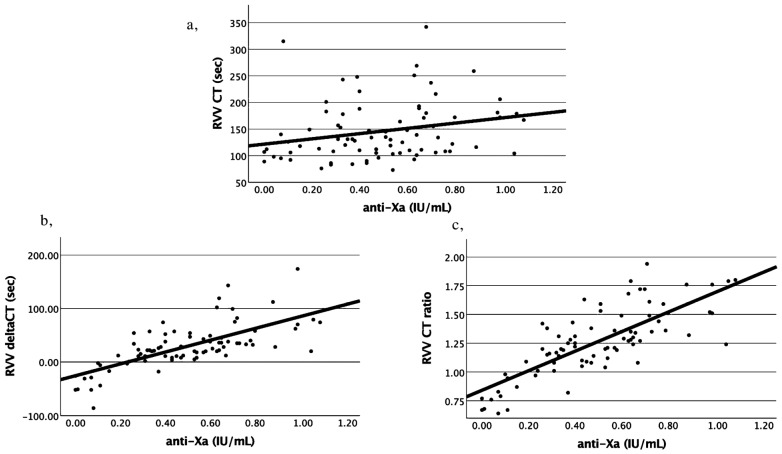
Bivariate correlations between anti-Xa values and RVV CT (**a**), RVV deltaCT (**b**) and RVV CT ratio (**c**). RVV delta CT = RVV CT without polybrene—RVV CT with polybrene. RVV CT ratio = RVV CT without polybrene/RVV CT with polybrene. RVV CT and deltaCT are given in seconds; RVV CT ratio has no dimension. Anti-Xa values are given in IU/mL. Pearson correlation coefficients and *p*-values are as follows: RVV CT (**a**) r = 0.240, *p* = 0.032; RVV deltaCT (**b**) r = 0.699, *p* < 0.001; RVV CT ratio r = 0.774, *p* < 0.001.

Correlations between the basic VET parameters (i.e., alfa angle, CFT, A5, A10, A20, MCF) and anti-Xa values were not significant, except for RVV CT, which had a very weak but statistically significant correlation (r = 0.240, *p* = 0.032) (Figure 2a). Both the RVV delta CT and RVV CT ratio values showed significant, stronger positive correlation with anti-Xa values (r = 0.699, *p* < 0.001 and r = 0.774, *p* < 0.001, respectively) (Figure 2b,c). Based on these results, the RVV CT ratio was selected for further analysis.

In healthy volunteers (median age and [range] in years: 29.5 [23–50]; 6 females and 4 males) without enoxaparin treatment, the presence of polybrene consistently prolonged RVV clotting times, reducing RVV CT ratios below one. The mean RVV CT without polybrene, mean RVV CT with polybrene and mean RVV CT ratio of healthy volunteers were 72.4 s (SD: 9.36), 101.1 s (SD:22.2) and 0.73 (SD: 0.10), respectively. The same phenomenon was observed in the group of patients with low anti-Xa values, mainly below 0.2 IU/mL.

**Figure 3:**
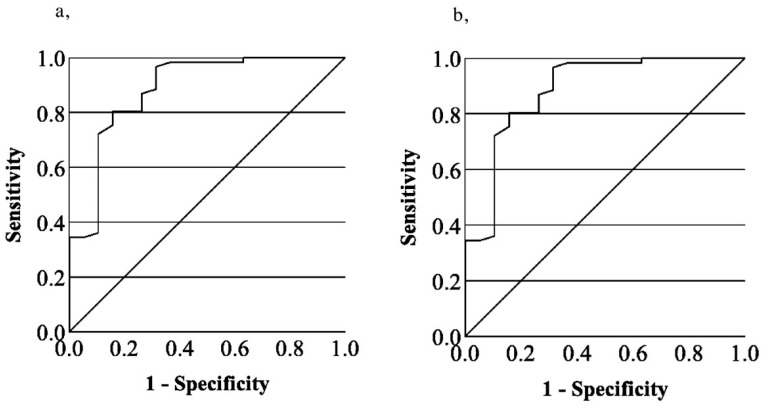
Receiver operating characteristics (ROC) curves for RVV CT ratio and anti-Xa cut off values of 0.3 IU/mL (**a**) and 0.6 IU/mL (**b**). AUC for 0.3 IU/mL anti-Xa was 0.885 (95% CI 0.789 to 0.981), *p* < 0.001. AUC for 0.6 IU/mL anti-Xa was 0.870 (95% CI 0.794 to 0.947), *p* < 0.001.

In patients’ group, to separate the low, medium and higher levels of enoxaparin, an-ti-Xa values of 0.3 and 0.6 IU/mL were selected (Figure 3). Based on ROC analysis we des-ignated RVV CT ratios of 1.02, 1.23 and 1.6 as the best cut-off values for separating anti-Xa ranges below and above 0.3 and 0.6 IU/mL, respectively. The distributions of patients in relation to RVV CT ratio and anti-Xa activity are shown in Table 2. The sensitivity, specificity and positive and negative predictive values for the respective anti-Xa and RVV CT limits are summarized in Table 3.

## 4. Discussion

Here, we present a possible approach: a modified POC method for rapid semiquantitative detection of enoxaparin.

Previous reports have suggested that ROTEM is not suitable for monitoring and detecting the effect of LMWH [10,17]. Consequently, the studies and case reports available on the subject are mainly related to the usage of TEG. Citrated blood of healthy individuals spiked ex vivo by LMWH showed a dose-dependent prolongation of the R values in tests performed by TEG [24,25,26]. However, the lack of activators in tests resulted in an undesirable increase in reaction time that was not compatible with a POC test [24,25]. Furthermore, in real patient population, the results were controversial and inconsistent with in vitro studies, failing to demonstrate a strong correlation between the anti-Xa activity and any standard TEG parameters including the R value [18,19,20]. It is a general observation that the positive results of in vitro experiments and studies involving healthy volunteers are not reflected in everyday clinical practice. Further attempts were made by the use of heparin neutralisers to increase the sensitivity of TEG to the effect of LMWH. The change in R value measured in the absence and presence of heparinase, the so-called delta R, showed a good correlation with anti-Xa activity. On the other hand, due to the observed considerable interindividual variability in delta R, it seemed not possible to estimate the value of anti-Xa [27]. There are a few case reports in which a residual LMWH effect was demonstrated successfully based on a significantly decreasing R-value in response to in vitro added heparinase, but without the quantification of LMWH [28,29,30].

Based on the lessons learned from previous studies outlined above, it appears that a viscoelastometric test for quantification of LMWH should use both an activator and a heparin neutraliser and focus primarily on CT or R values.

We examined 80 patients under enoxaparin treatment using the application of the commercially available ClotPro viscoelastometric machine and its RVV-test containing Russell’s viper venom as a direct FX activator. Polybrene was selected as a heparin neutraliser.

Theoretically, measurements from the RVV-test of ClotPro can eliminate all influencing factors on CT prior FX in the coagulation cascade. Among the ClotPro viscoelastometric reagents, the RVV-test was found to be the most sensitive to the presence of LMWH [21,31]. In addition, the RVV-test was proved as a useful method for the detection of FXa inhibitory effect of direct oral anticoagulants [32]. Therefore, it seemed to be the best choice among viscoelastometric reagents for quantifying enoxaparin with dominant FXa inhibition.

To improve the applicability of the ClotPro RVV-test for enoxaparin assessment, we used a heparin neutraliser. As compared to heparinase, polybrene has the advantages of immediate action, low cost and greater efficiency. The highly positively charged polybrene can bind to negative charges of LMWH and completely neutralises its antithrombotic effect confirmed by measurements of anti-Xa activity and thrombin generation [33,34,35]. Consequently, the difference between VET parameters detected in the lack of and in the presence of polybrene is expected to reflect the specific anticoagulant effect of LMWH. On the other hand, the in vitro anticoagulant effect of polybrene in the absence of heparin has long been known [36]. In our study, polybrene also prolonged the RVV clotting time resulting in a RVV CT ratio lower than 1.0 in the group of healthy volunteers and in the presence of only a small amount of enoxaparin in the group of patients. We applied polybrene in a high concentration; therefore, it can be assumed that the free polybrene in the absence of enoxaparin can interfere with the action of negatively charged phospholipids and other molecules involved in the coagulation.

Among the standard coagulation assays and the measured and calculated VET parameters, the RVV CT ratio showed the best, statistically significant correlation with anti-Xa activity. Subsequently, we used it to estimate the amount of enoxaparin.

Peak anti-Xa values are generally measured in the ranges of 0.5–1.2 IU/mL in cases of therapeutic administration of LMWH. At prophylactic doses of LMWH, peak anti-Xa values are usually in the range of 0.2–0.5 IU/mL [10,12,37]. The anti-Xa activities of 0.3 and 0.6 IU/mL may be considered reasonable and appropriate cut-off values for everyday clinical practice. Below 0.3 IU/mL, it is considered as a low range, while intermediate range is between 0.3 and 0.6 IU/mL and above 0.6 IU/mL, the therapeutic and overdose ranges of enoxaparin are estimated.

Based on our investigation of 80 real-life patients, the ROC curve analysis suggested the use of RVV CT ratios of 1.02, 1.23 and 1.6 to separate these anti-Xa ranges with acceptable sensitivity and specificity. If the RVV CT ratio is below or above 1.23, the anti-Xa activity is suggested to be below 0.6 IU/mL or above 0.3 IU/mL with high certainty (predictive values of 0.97 and 0.95), respectively. In the second step, further differentiation is possible if the RVV CT ratio is measured below 1.02 or above 1.6. In these cases, the measured anti-Xa values are below 0.3 IU/mL or above 0.6 IU/mL with high probability and predictive values of 0.87 and 0.91, respectively (Table 2 and Table 3; Figure 1). No further distinction could be made among anti-Xa values in the range below 0.3 IU/mL and this was found to be the lowest well-distinguishable anti-Xa value according to the RVV CT ratio. It should be emphasized that these data are based on a single-centre study and measurements with a semi-automatic POC device, which limits the generalizability of our findings. However, using this method, novel and local RVV CT ratio cut-off values may be determined, if necessary, even for other anti-Xa values, depending on the local conditions and the purpose of the clinical application.

**Figure 4:**
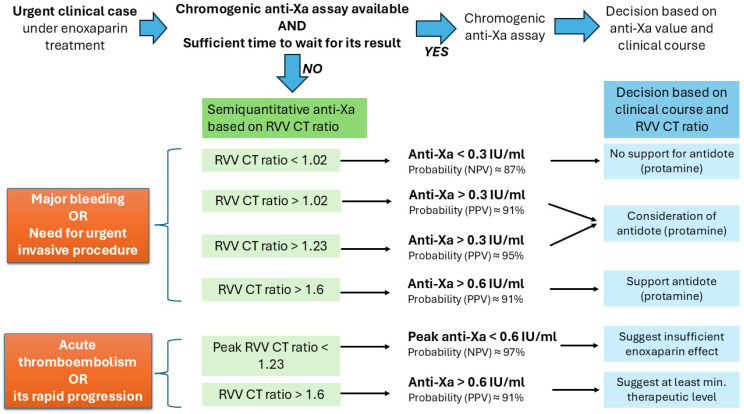
Decision support algorithm in urgent clinical cases under enoxaparin therapy in the authors’ institute. Possible approach in acute clinical situations of patients receiving heparin therapy using RVV CT ratio measured by ClotPro. It should be emphasized that this approach is based on a single-centre study and measurements with a semi-automatic POC device, which limits the generalizability of cut-off values. However, using the method outlined in the text, novel and local RVV CT ratio cut-off values may be determined if necessary, even for other anti-Xa values, depending on the local conditions and the purpose of the clinical application.

In urgent cases, when chromogenic anti-Xa assay is not available or there is no time to wait for its result, these limits can help guide key decisions. Protamine sulfate is the commonly used partial antidote of LMWHs exerting its effect by electrostatic binding to anionic heparins [38,39,40,41]. However, protamine remaining in excess of an oversized dose provides an anticoagulant effect by inhibition of coagulation, enhanced fibrinolysis and impairment of platelet function [39]. Thus, unnecessary use or overdose of protamine should be avoided. Therefore, in our institute, if the RVV CT ratio is above 1.23 (anti-Xa > 0.3 IU/mL), especially if it is above 1.6 (anti-Xa > 0.6 IU/mL), a meaningful enoxaparin effect can be assumed and the administration of an antidote should be considered in case of major bleeding. In the acute phase of arterial or venous thromboembolism treated with enoxaparin, an RVV CT ratio below 1.23 (anti-Xa < 0.6 IU/mL) may suggest insufficient enoxaparin effects. In case of urgent major surgery of a patient receiving enoxaparin, reasonable delay of surgery or the administration of an antidote is supported if the RVV CT ratio is above 1.6 (anti-Xa > 0.6 IU/mL), while no meaningful enoxaparin effect is expected and protamine sulfate may be avoided if the RVV CT ratio is below 1.02 (anti-Xa < 0.3 IU/mL) (Figure 4). Each case undoubtedly requires an individual assessment and the RVV CT ratio should always be evaluated together with the clinical course.

With proper organization and the use of certain techniques, such as the rapid centrifugation method and the reduction in incubation time, the turnaround time (TAT) of anti-Xa determination can be significantly reduced [13,14]. In an earlier paper by Seiffge et al., the median TAT for the rapid determination of anti-Xa using the chromogenic method was 34 min (IQR 29–65 min) [14]. However, the availability of the rapid anti-Xa determination in a 24/7 system is limited. In our study, the TAT for determining the RVV CT ratio was 5 min of sample preparation and pipetting (median time 302 s; IQR 293–319 s), plus the longer duration of the paired RVV clotting times measured in the presence and absence of polybrene (median time 132 s; IQR 107–174 s without polybrene; median time 114 s; IQR 88–144 s with polybrene) (Figure 1). The ClotPro provides the available data immediately on the screen in real time. In this way, despite the 60 min running time, the clotting time as the first measured parameter, as well as the RVV CT ratio, are available within a few minutes. Furthermore, a POC test can also save the transport time of the sample to the central laboratory.

It should be noted that our method is not suitable for predicting clinical events such as bleeding or thrombosis during enoxaparin treatment. The chromogenic method is the first choice to determine anti-Xa activity of LMWH if we have sufficient time and it is available, providing more accurate and quantitative results. However, semiquantitative determination of anti-Xa activity based on the RVV CT ratio providing results within 8–10 min may be very useful in situations associated with serious time factors such as life-threatening bleeding, need for urgent invasive procedure associated with high risk of bleeding or acute thromboembolism and its rapid progression when an immediate clinical decision and action has to be made under enoxaparin therapy.

The study we conducted has some limitations. The accuracy and reproducibility of viscoelastometric tests are somewhat lower than those of sophisticated and conventional laboratory methods. In the case of the ClotPro device, the coefficient of variation for CT was found between 2.5 and 7.2% in the presence of different activators [42]. According to the package insert provided by the manufacturer for the ClotPro RVV test, the coefficient of variance was found to be 4.5% for the parameter of RVV CT. Accordingly, the results should always be carefully interpreted in the context of each individual clinical setting. Viscoelastometric parameters measured by different viscoelastometric devices and their reagents are not interchangeable. The results of our study are applicable only for RVV-tests performed by ClotPro. In addition, we examined only patients receiving enoxaparin. Other LMWH preparations were not the subject of our study.

Our method requires some pipetting; however it can still be used as a POC test in emergency situations. Learning the correct pipetting technique and ClotPro use are necessary for accurate measurement. We presented a single-centre study limiting the generalizability of our findings. Each institute needs to carry out confirmatory tests on their own ClotPro device, under local conditions, by their own staff, to verify the local applicability of the method and cut-off values due to potential uncertainties arising from semi-automatic POC measurement.

Tests using RVV-containing reagents may be sensitive to the presence of other anticoagulants and lupus anticoagulant [43]. Artificial or accidental contamination of the sample with heparin or the presence of other anticoagulants such as direct oral anticoagulants, vitamin K antagonists, etc., should be avoided and excluded as it can lead to false results. Although the use of heparin-neutralising polybrene and the calculation of the RVV CT ratio theoretically eliminates confounding factors such as the presence of lupus anticoagulant, we do not have sufficient experience with the measurement in this condition.

## 5. Conclusions

In conclusion, we have established a point-of care method based on viscoelastometric measurements using polybrene and Russell’s viper venom that can provide rapid, semiquantitative information on the effect of enoxaparin and the expected anti-Xa activity within 10 min in real clinical situations. It can separate cases with low, medium and higher anti-Xa activities with good predictive values, sensitivity and specificity. In this regard, it may support more rapid and objective clinical decisions to be made in cases of life-threatening bleeding or acute thromboembolism, as well as in the perioperative period of urgent surgeries under enoxaparin therapy, when chromogenic anti-Xa measurement is not available or there is no time to wait for its result.

## Figures and Tables

**Table 1:** Clinical characteristics, anti-Xa and ClotPro RVV assay values and preanalytic time of the 80 included patients. Data are presented as median [IQR, range] for anti-Xa, RVV CT without and with polybrene, RVV CT ratio and preanalytic time, or as mean ± SD for age. RVV CT ratio = RVV CT without polybrene/RVV CT with polybrene; ICU, intensive care unit.

	Total *n* = 80
Sex (male), *n* (%)	52 (65)
Age (mean ± SD), years	65 ± 15
Prophylactic anticoagulation in ICU, *n* (%)	33 (41)
Acute medical illness, n (%)	22 (27)
Perioperative settings, n (%)	11 (14)
Therapeutic anticoagulation, *n* (%)	47 (59)
Venous thromboembolism, n (%)	27 (34)
Atrial fibrillation, n (%)	20 (25)
Anti-Xa (IU/mL)	0.40 [0.24 to 0.64, 0.01 to 1.06]
RVV CT without polybrene (s)	132 [107 to 174, 73 to 342]
RVV CT with polybrene (s)	114 [88 to 144, 60 to 401]
RVV CT ratio	1.25 [1.08 to 1.42, 0.64 to 1.94]
Preanalytic time (sample preparation and pipetting) (s)	302 [293 to 319, 275 to 342]

**Table 2:** Patient distribution according to anti-Xa and RVV CT ratio values: (A) anti-Xa 0.6 IU/mL and RVV CT ratio 1.23 cut-off values; (B) anti-Xa 0.3 IU/mL and RVV CT ratio 1.23 cut-off values; (C) anti-Xa 0.3 IU/mL and RVV CT ratio 1.02 cut-off values; (D) anti-Xa 0.6 IU/mL and RVV CT ratio 1.6 cut-off values.

		**RVV CT Ratio 1.23**						**RVV CT Ratio 1.23**		
		Below	Above	Total patients				Below	Above	Total patients
**anti-Xa** **0.6 IU**/mL	Below	36	15	51		**anti-Xa** **0.3 IU**/mL	Below	17	2	19
	Above	1	28	29			Above	20	41	61
	Total Patients	37	43	80			Total patients	37	43	80
**2A**						**2B**				
		**RVV CT ratio 1.02**						**RVV CT ratio 1.6**		
		Below	Above	Total patients				Below	Above	Total patients
**anti-Xa** **0.3 IU**/mL	Below	13	6	19		**anti-Xa** **0.6 IU**/mL	Below	50	1	51
	Above	2	59	61			Above	19	10	29
	Total patients	15	65	80			Total patients	69	11	80
**2C**						**2D**				

**Table 3:** Specificity, sensitivity and positive and negative predictive values of paired RVV CT ratio and anti-Xa cut-off values. Negative predictive value means the probability that both the RVV CT ratio determined by viscoelastometric device and the anti-Xa value measured by chromogenic method are below the paired RVV CT ratio and anti-Xa cut-off values, respectively. Positive predictive value means the probability that both the RVV CT ratio determined by viscoelastometric device and the anti-Xa value measured by chromogenic method are above the paired RVV CT ratio and anti-Xa cut-off values, respectively. Values are calculated based on the number of patients classified into the different groups according to their anti-Xa and CT ratio values demonstrated in Table 2.

RVV CT Ratio	Anti-Xa Activity	Sensitivity	Specificity	Positive Predictive Value	Negative Predictive Value
<1.02	<0.3 IU/mL	0.97 (0.946–0.994)	0.68 (0.665–0.695)	0.91 (0.888–0.932)	0.87 (0.849–0.891)
>1.23	>0.3 IU/mL	0.67 (0.656–0.684)	0.89 (0.868–0.912)	0.95 (0.927–0.973)	0.46 (0.453–0.467)
<1.23	<0.6 IU/mL	0.97 (0.946–0.994)	0.71 (0.694–0.726)	0.65 (0.637–0.663)	0.97 (0.946–0.994)
>1.60	>0.6 IU/mL	0.34 (0.326–0.354)	0.98 (0.956–1.00)	0.91 (0.888–0.932)	0.72 (0.704–0.736)

## Data Availability

Data are contained within the article. Additional supporting data are available from the corresponding author on request.
